# Supplementation with sodium butyrate improves growth and antioxidant function in dairy calves before weaning

**DOI:** 10.1186/s40104-020-00521-7

**Published:** 2021-01-04

**Authors:** Wenhui Liu, A. La Teng Zhu La, Alexander Evans, Shengtao Gao, Zhongtang Yu, Dengpan Bu, Lu Ma

**Affiliations:** 1 Institute of Animal Science, State Key Laboratory of Animal Nutrition, Chinese Academy of Agricultural Sciences, No. 2 Yuanmingyuan West Road, Beijing, 100193 People’s Republic of China; 2 School of Agriculture & Food Science, University College Dublin, Belfield, Dublin 4, Ireland; 3 Department of Animal Sciences, The Ohio State University, Columbus, OH 43210 USA; 4 Joint Laboratory on Integrated Crop-Tree-Livestock Systems of the Chinese Academy of Agricultural Sciences (CAAS), Ethiopian Institute of Agricultural Research (EIAR) and World Agroforestry Center (ICRAF), Beijing, 100193 People’s Republic of China

**Keywords:** Antioxidant activity, Calf, Immune function, Sodium butyrate

## Abstract

**Background:**

There is increasing research interest in using short-chain fatty acids (SCFAs) including butyrate as potential alternatives to antibiotic growth promoters in animal production. This study was conducted to evaluate the effects of supplementation of sodium butyrate (SB) in liquid feeds (milk, milk replacer, and the mixture of both) on the growth performance, rumen fermentation, and serum antioxidant capacity and immunoglobins in dairy calves before weaning. Forty healthy female Holstein calves (4-day-old, 40 ± 5 kg of body weight) were housed in individual hutches and randomly allocated to 1 of 4 treatment groups (*n* = 10 per group) using the RAND function in Excel. The control group was fed no SB (SB0), while the other three groups were supplemented with 15 (SB15), 30 (SB30), or 45 (SB45) g/d of SB mixed into liquid feeds offered. The calves were initially fed milk only (days 2 to 20), then a mixture of milk and milk replacer (days 21 to 23), and finally milk replacer only (days 24 to 60).

**Results:**

The SB supplementation enhanced growth and improved feed conversion into body weight gain compared with the SB0 group, and the average daily gain increased quadratically with increasing SB supplementation. No significant effect on rumen pH; concentrations of NH_3_-N, individual and total VFAs; or acetate: propionate (A:P) ratio was found during the whole experimental period. Serum glutathione peroxidase activity increased linearly with the increased SB supplementation, while the serum concentration of maleic dialdehyde linearly decreased. Serum concentrations of immunoglobulin A, immunoglobulin G, or immunoglobulin M were not affected by the SB supplementation during the whole experimental period.

**Conclusions:**

Under the conditions of this study, SB supplementation improved growth performance and antioxidant function in pre-weaned dairy calves. We recommended 45 g/d as the optimal level of SB supplementation mixed into liquid feeds (milk or milk replacer) to improve the growth and antioxidant function of dairy calves before weaning.

## Introduction

The digestive physiology of calves changes dramatically in the first months of their lives, and the transition from a monogastric to the functional ruminant digestive system is fraught with challenges [1]. The development of the gastrointestinal (GI) tract, especially the rumen, is one of the most important steps profoundly affecting the nutritional status and growth performance of young dairy calves and lactation performance during their adult lives. A successful development of the GI tract can decrease disease susceptibility and mortality and improve the nutrition of dairy cows and the profitability of dairy producers [2]. The physiology of GI development is complex [3] and can be aided by some antibiotics used for growth promotion [4]. However, extensive use of antibiotics increases the development of antibiotic resistance in animals and its dissemination, posing a threat to public health [5–7]. Non-antibiotic alternatives are needed as the use of antibiotics decreases to comply with government policy or meet consumer or societal demands.

Butyric acid products (including their acid and salt forms) have the potential to replace certain antibiotic growth promoters as feed additives [8, 9]. Supplementation with butyric acid, for example, has been shown to stimulate animal growth by enhancing the proliferation, differentiation, and function of gut tissues [10]. It was postulated that SB supplementation might improve performance in calves through modulating the rumen and hindgut microbiota, particularly the cecal microbiota [11]. Studies have shown that SB can promote the growth of calves and enhance feed digestion and nutrient absorption in the small intestines [12], decrease inflammation, improve the antioxidant and immune capacity, increase feed intake and daily gain, and improve feed conversion ratio in piglets and calves [12–16]. Several studies have also evaluated SB for its ability to promote calf GI development and improve nutrient absorption [17, 18].

However, the outcome of SB supplementation to promote calf growth and health has been discrepant. For example, one study showed that supplementation with SB at 0.3% to 1% of dry matter (DM) in milk replacer (MR) increased ADG in calves before and after weaning [15]. In another study [19], SB increased average daily gain (ADG) and final BW in heifers when supplemented with increasing SB from 0 to 0.75 g/kg of BW. However, Wanat et al. [20] reported conflicting results that even at 0.3%, 0.6%, or 0.9% of DM, microencapsulated SB added to starter mixture reduced the growth performance in calves, including linear decreases in ADG and BW in a dose-dependent manner. Ślusarczyk et al. [21] showed that SB was well tolerated and it improved growth performance when supplemented at 1% and 3% of DM mixed in starter before weaning (56 days of age). However, it was found that SB supplementation at 3% of DM reduced feed intake despite a positive effect on calf growth and nutrient utilization. Ferreira et al. [22] reported that the inclusion of SB (150 g/kg DM), calcium propionate (150 g/kg DM), or sodium monensin (30 mg/kg DM) in a starter feed did not improve animal growth performance, both before and after weaning. Górka et al. [23] showed that SB added into MR (0.3% of DM) positively affected BW gain, health, some metabolic intermediates in calves, and rumen development indirectly, while SB supplemented to a starter mixture (0.6% of DM) stimulated rumen development directly. Based on their results, Górka et al. [23] recommended the addition of SB either into MR or starter in rearing calves. However, in a later study, the same authors showed that this effect was more profound when SB was mixed into MR than when SB was mixed into the starter mixture before weaning, although both routes of SB supplementation could enhance the development of the small intestine [24]. Moreover, most of the studies on SB supplementation have been focused on how it might affect feed intake, rumen fermentation, and animal growth including rumen tissue growth. However, the effects of supplementation with SB on antioxidant capacity or immune function in calves have not been determined. Therefore, the present study aimed to investigate the effects of SB supplementation at different levels (mixed into milk and/or MR) on the growth performance, rumen fermentation, health, antioxidant capacity, and immune function in calves before weaning and to determine the optimal level of dietary supplementation of SB during the early period of raising dairy calves.

## Materials and methods

### Animals, treatments, and management

This study was started in July 2018 on a commercial dairy farm located in the City of Dongying, Shandong Province, China. The Institutional Animal Care and Use Committee of the Institute of Animal Sciences at the Chinese Academy of Agricultural Sciences approved all the experimental procedures (protocol no. IAS 20180115). Forty healthy female Holstein calves (4-day-old, 40 ± 5 kg of BW) born within 1 w on that dairy farm were recruited and separated from their dam immediately after birth. They were placed in individual south-facing Calf-Tel hutches (Hampel Corp., Germantown, WI) approximately 1.5 m apart. The hutches were bedded with sand and placed on a sand base. The calves were randomly allocated to 1 of 4 treatment groups (*n* = 10 calves per group) using the RAND function of Excel. The control group was fed no SB (SB0), while three treatment groups were fed SB (98.5% purity, Enkefu Co. Ltd., Beijing) mixed into liquid feeds (milk and then MR) at 15 (SB15), 30 (SB30), or 45 (SB45) g/d per calf. The doses of SB supplementation were based on the study of Slusarczyk et al. [21] who fed calves SB at 2.2, 7.3, or 22 g/d before weaning.

Prior to the feeding experiment, all calves were fed 4 L of colostrum within 1 h after birth and then two more feedings of colostrum 6 h (2 L) and 18 h (1 L) later. All calves were fed per the feeding regimen of the dairy farm. Specifically, the calves were fed only milk from 2 to 20 days of age. From 21 to 23 days of age, the calves were fed a mixture of milk and MR (Eurolac Blue, Netherlands) at different milk:MR volumetric ratio: 75% milk and 25% MR at 21 days of age, 50% milk and 50% MR at 22 days of age, 25% milk and 75% MR at 23 days of age. All the calves were fed only MR from 24 to 60 days of age (end of the experiment). The MR was dissolved in water to a final total solid content of 17.86%. All calves were fed the liquid feeds (milk, MR, and the mixture of both) using individual open buckets twice daily at 07:00 h and 15:00 h according to the following feeding regimen: 2.5 L/meal from 2 to 7 days of age, 3 L/meal from 8 to 10 days of age, 3.5 L/meal at 11 days of age, 4 L/meal at 12 days of age, 4.5 L/meal from 13 to 30 days of age, 6.5 L/meal from 31 to 50 days of age, 5.5 L/meal at 51 days of age, 4.5 L/meal at 52 days of age, and then the allowance of the previous day with 1 L decrement per day (0.5 L/meal) until weaning at 60 days of age. The preset amounts of SB and liquid feed allowance were added together to feeding buckets and manually stir-mixed to dissolve the SB prior to each feeding. Each of the daily doses of SB was divided into 2 equal portions and fed in the morning and the afternoon. A pelleted starter feed (≥ 24.0% CP declared by the manufacturer, Rubeiyou8100, Yuan Xing Co., Ltd., China) was offered to the calves once daily after the liquid feeding in the morning from 4 days of age onward. When the starter ort was less than 20 g, an additional 100 g of starter was added the next day to ensure adequate starter was available all the time. The chemical composition of the experimental feeds (milk, MR, and starter) is presented in Table 1.

**Table 1:** Chemical composition of experimental feeds

Items	Milk	Milk replacer^a^	Starter^a, b^
Ingredients, %			
Wheat bran	–	–	5.92
Steam-flaked corn	–	–	40.59
Cane molasses	–	–	1.53
Soybean meal	–	–	20.63
Extruded soybean	–	–	6.05
Canola meal	–	–	11.78
Corn gluten	–	–	2.46
Wheat shorts	–	–	7.12
Calves starter premix^b^	–	–	3.93
Total	–	–	100.00
DM, %	–	96.06	97.23
CP, %	–	22.49	25.94
EE, %	–	9.35	3.01
Ash, %	–	7.16	6.47
NDF, %	–	0.78	16.03
ADF, %	–	0.54	7.00
Ca, %	–	1.15	0.94
P, %	–	0.97	0.66
Density, g/L	1030.50	–	–
Milk protein, %	3.50	–	–
Milk fat, %	3.88	–	–
Total solid, %	12.93	–	–
DM, %	12.30	–	–
Lactose, %	4.36	–	–

^a^ on DM basis

^b^ The premix provided per kg of the starter was as follows: vitamin A 13,050 IU, vitamin D 3262 IU, vitamin E 260.997 IU, Fe 116.817 mg, Cu 19.621 mg, Mn 48.516 mg, Zn 74.603 mg, Se 0.746 mg, I 1.343 mg, Co 0.966 mg

### Calf growth measurement, sample collection, and analysis

Body length, BW, wither height, and heart girth were recorded at the beginning, 4, 14, 28, 42, and 60 days of age before the morning feeding. Average daily gain was calculated over the above time intervals and the entire experiment period. Intake of starter feed was recorded daily and for each calf at 09:00 h, and intake of liquid feed was recorded twice daily for each calf. Total dry matter intake (DMI) was calculated based on the consumption of the liquid feed and starter for each calf. Feed-to-gain (F:G) ratio was calculated as the ratio of total DMI to ADG.

Rumen fluid (about 25 mL) was collected from each calf 2 h after the morning feeding of the liquid feed at 14, 28, and 60 days of age via a flexible esophageal tube (2 mm wall thickness, 6 mm i.d.) and a pump (Anscitech Co. Ltd., Wuhan, Hubei, China). The first 5 mL was discarded to avoid contamination with saliva. The individual rumen fluid samples were squeezed through 4 layers of cheesecloth; the pH was measured immediately, and then 6 mL each of strained fluid was acidified with 3 mL of 0.5 mol/L HCl and frozen at − 20 ℃ for ammonia nitrogen (NH_3_-N) analysis [25]. A 4-mL aliquot from each sample was prepared for volatile fatty acid (VFA) analysis using gas chromatography as described by Erwin et al. [26].

Blood samples were taken from the external jugular vein of each calf 2 h after the morning feeding of the liquid feed at 14, 28, and 60 days of age. At each collection, a duplicate 10 mL of blood samples were placed into tubes containing no additives. Serum was prepared by centrifugation at 3000×*g* for 15 min at 4 ℃ and then stored at − 20 ℃ until analysis. Activities of glutathione peroxidase (GSH-Px), superoxide dismutase (SOD), and concentration of maleic dialdehyde (MDA) were analyzed using respective commercial kits (Nanjing Jian Cheng Bioengineering Institute, Nanjing, China) as described previously [27]. The inter-assay CVs of SOD, MDA, and GSH-Px were 1.57, 3.28, and 3.21, respectively, and the intra-assay CVs of SOD, MDA, and GSH-Px were 3.64, 4.29, and 4.51, respectively. The serum concentrations of immunoglobulin (Ig) A (IgA), IgG, and IgM were determined using ELISA kits (F4042-A, F3995-A, and F6685-A, respectively) (Shanghai Panke Industrial Co., Shanghai, China). The inter-assay CVs of IgG, IgA, and IgM were 7.30, 6.89, and 5.34, respectively, and the intra-assay CVs of IgG, IgA, and IgM were 8.69, 9.23, and 8.06, respectively.

Samples of MR and starter were collected once per week, and the content of DM, crude protein (CP), ash, ether extract (EE), acid detergent fiber (ADF), neutral detergent fiber (NDF), calcium (Ca), and phosphorus (P) were determined as described in a previous study [28]. The content of protein, fat, lactose, total solids, and non-fat solids of the milk was determined with the mid-infrared procedures using a Milk Oscan Minor machine (MilkoScan Type 78,110; Foss Electric, Hillerød, Denmark).

### Statistical analysis

Statistical analysis was performed using SAS v. 9.4 (SAS Institute, Cary, NC) with all data tested first for normality. Linear, quadratic, and cubic polynomial contrasts were tested using the CONTRAST statement of SAS. The statistical model for ADG, liquid feed DM intake, starter DMI, total DMI, and F:G ratio included calf as random effect, treatment, day, and interaction between treatment and day as fixed effects, day as repeated effect, initial BW and parity of the dams as covariate. The statistical model for rumen fermentation characteristics, MDA, CAT, GSH-Px, SOD, IgA, IgM, and IgG included calf as random effect, treatment, day, and interaction between treatment and day as fixed effects, day as repeated effect, and parity of the dams as covariate. The statistical model for final BW, withers height, body length, and heart girth included treatment as fixed effects with initial BW, initial withers, initial body length, initial heart girth and parity of the dams as covariate. The covariance structure was autoregressive based on Akaike’s Information Criterion (AIC) and Schwarz’s Bayesian Criterion (SBC).

Differences were considered as statistically significant when *P* < 0.05, and a tendency was considered when 0.05 ≤ *P* ≤ 0.10. Statistical power analysis was performed with α = 0.05 and power = 0.80 using PROC POWER procedure of SAS (SAS Institute Inc., Cary, NC), and with 10 calves per treatment group, a 10% difference between treatment means for most variables would be found with a power of 80% or greater.

## Results

We determined DMI, ADG, F:G ratio, rumen fermentation characteristics, serum concentrations of IgA, IgG, and IgM, antioxidant enzyme activities, and MDA concentration at different ages of the animals (data not shown). Except for F:G ratio, no significant treatment × day interaction was noted (Tables 2, 3, 4 and 5). Thus, we only reported the overall effect of SB supplementation of the entire experiment.

**Table 2:** Growth performance of calves fed different levels of sodium butyrate

Items	Treatment^a^				SEM	*P*-value				
	SB0	SB15	SB30	SB45		Linear	Quadratic	Cubic	Trt^b^ × Day	Day
Initial BW, kg	40.6	38.8	39.7	39.4	0.38					
Final BW, kg	90.6	93.4	92.2	92.8	0.58	0.441	0.448	0.376		
Gain of BW, kg	50.0	54.6	52.5	53.4	0.61	1.000	1.000	1.000		
ADG, kg/d	0.83	0.90	0.89	0.88	0.02	0.086	**0.034**	0.230	0.351	<.0001
Total DMI ^c^, g/d	1201.8	1202.9	1191.7	1186.9	3.90	0.545	0.886	0.843	0.988	<.0001
Starter DMI, g/d	123.6	122.2	114.9	113.8	1.59	0.692	0.994	0.897	0.963	<.0001
Liquid diet DMI, g/d	1078.2	1080.7	1076.8	1073.1	1.59	0.165	0.317	0.650	0.494	<.0001
F:G ratio ^d^	1.45	1.33	1.34	1.35	0.06	0.061	0.354	0.401	<.001	<.0001
Initial withers height, cm	76.6	74.4	74.7	75.0	0.49					
Final withers height, cm	93.3	92.9	93.7	93.2	0.17	0.882	0.913	0.442		
Gain of withers height, cm	16.7	18.5	19.0	18.2	0.35	0.478	0.079	0.708		
Initial body length, cm	70.8	68.9	69.7	69.6	0.39					
Final body length, cm	90.9	92.3	92.4	91.8	0.34	0.254	0.082	0.782		
Gain of body length, cm	20.1	23.4	22.7	22.2	0.52	0.054	0.061	0.599		
Initial heart girth, cm	82.3	83.2	81.8	83.1	0.33					
Final heart girth, cm	105.0	105.1	104.6	104.3	0.18	0.287	0.779	0.742		
Gain of heart girth, cm	22.7	22.9	22.8	21.2	0.38	0.124	0.368	0.198		

Means (*n* = 10 per treatment group) with different superscripts in a row differ significantly (*P* < 0.05). Tendency *P* values are underlined, and significant *P* values are bolded

^a^ SB0, SB15, SB30, and SB45: 0, 15, 30, and 45 g of sodium butyrate per day, respectively, were supplemented

^b^ *Trt* Treatment

^c^ Total DMI = starter DMI + liquid feed DMI

^d^ Feed-to-gain ratio was calculated by total DMI to ADG

**Table 3:** Rumen fermentation characteristics of calves fed different levels of sodium butyrate

Items	Treatment^a^				SEM	*P*-value				
	SB0	SB15	SB30	SB45		Linear	Quadratic	Cubic	Trt^b^ × Day	Day
pH	6.42	6.59	6.41	6.32	0.06	0.221	0.134	0.258	0.790	<.0001
NH_3_-N, mg/dL	18.13	17.96	16.50	18.22	0.40	0.865	0.533	0.501	0.723	0.032
Acetic acid, mmol/mL	27.86	27.64	29.83	29.77	0.59	0.377	0.967	0.577	0.718	<.0001
Propionic acid, mmol/mL	29.01	28.40	27.62	30.90	0.70	0.601	0.340	0.629	0.143	<.0001
Isobutyric acid, mmol/mL	0.61	0.62	0.64	0.52	0.03	0.375	0.313	0.569	0.323	0.001
Butyric acid, mmol/mL	7.27	9.42	9.73	9.39	0.57	0.120	0.172	0.757	0.417	<.0001
Isovaleric acid, mmol/mL	0.69	0.75	0.70	0.63	0.02	0.576	0.497	0.880	0.402	0.002
Valeric acid, mmol/mL	2.02	1.82	2.25	2.13	0.09	0.580	0.906	0.358	0.525	<.0001
Total VFA, mmol/mL	67.63	68.23	70.91	72.88	1.22	0.359	0.874	0.882	0.143	<.0001
Acetic: Propionic	1.21	1.23	1.32	1.190	0.03	0.946	0.244	0.270	0.409	<.0001

^a^ SB0, SB15, SB30, and SB45: 0, 15, 30, and 45 g of sodium butyrate per day, respectively, were supplemented

^b^ *Trt* Treatment (*n* = 10 per treatment group)

**Table 4:** Serum concentration of maleic dialdehyde (MDA) and activities of catalase (CAT), glutathione peroxidase (GSH-Px), and superoxide dismutase (SOD) in the calves fed different levels of sodium butyrate

Items	Treatment^a^				SEM	*P*-value				
	SB0	SB15	SB30	SB45		Linear	Quadratic	Cubic	Trt^b^ × Day	Day
SOD, U/mL	8.18	8.10	7.29	8.13	0.21	0.708	0.438	0.366	0.774	0.037
CAT, U/mL	3.98	5.00	4.68	4.50	0.21	0.454	0.108	0.370	0.304	<.0001
GSH-Px, U/mL	106.74	114.97	126.57	129.79	5.32	**< 0.001**	0.535	0.510	0.689	0.041
MDA, nmol/mL	2.53	2.16	2.17	1.95	0.12	**0.011**	0.613	0.374	0.817	<.0001

Means (*n* = 10 per treatment group) with different superscripts in a row differ significantly (*P* < 0.05). Significant *P* values are bolded

^a^ SB0, SB15, SB30, and SB45: 0, 15, 30, and 45 g of sodium butyrate per day, respectively, were supplemented

^b^ *Trt* Treatment (*n* = 10 per treatment group)

**Table 5:** Serum immunoglobin (Ig) concentration of calves fed different levels of sodium butyrate

Items	Treatment^a^				SEM	*P*-value				
	SB0	SB15	SB30	SB45		Linear	Quadratic	Cubic	Trt^b^ × Day	Day
IgA, mg/mL	0.72	0.74	0.71	0.73	0.01	0.939	0.963	0.513	0.231	0.510
IgG, mg/mL	5.82	5.73	5.44	5.73	0.08	0.630	0.450	0.503	0.607	0.241
IgM, mg/mL	0.20	0.19	0.19	0.20	< 0.01	0.680	0.115	0.906	0.637	0.813

^a^ SB0, SB15, SB30, and SB45: 0, 15, 30, and 45 g of sodium butyrate per day, respectively, were supplemented

^b^ *Trt* Treatment (*n* = 10 per treatment group)

### Growth performance and rumen fermentation

The effects of SB supplemented in liquid feeds on ADG, liquid feed DMI, starter DMI, total DMI (both liquid feed and starter), F:G ratio, BW, and body size measurements are presented in Table 2. The ADG increased quadratically with the increasing SB dose (*P* = 0.034), while the F:G ratio tended to decrease linearly with increasing SB dose (*P* = 0.061). The gain of withers height and body length tended to increase quadratically with the increasing SB dose (*P* = 0.079, *P* = 0.061). The ADG, total DMI, and starter DMI significantly increased (*P* < 0.01) biweekly (data not shown). The effects of day and treatment × day interaction on F:G ratios were significantly decreased (*P* < 0.01) over the whole experiment period (data not shown).

Rumen pH, NH_3_-N concentration, concentrations of individual and total VFAs, or acetate:propionate ratio were not affected (*P* > 0.05) by the SB supplementation during the whole experimental period (Table 3). No treatment × day interaction on any of these parameters was observed (*P* > 0.05). The pH and acetate: propionate ratio were significantly decreased (*P* < 0.01), while the concentrations of individual and total VFAs were significantly increased (*P* < 0.01) biweekly (data not shown). The concentration of NH_3_-N was increased before day 28, while decreased on day 60 (*P* < 0.05) (data not shown).

### Antioxidant capability and immunoglobulins in serum

The effects of the SB supplementation on the serum antioxidant capability in the pre-weaned calves are shown in Table 4. No significant effect of SB supplementation on the activities of serum SOD or CAT was found during the whole experimental period (*P* > 0.05), although the effects of day on these parameters were significant (*P* < 0.05). However, the serum GSH-Px activity was linearly increased (*P* < 0.05), while the serum MDA concentration linearly decreased (*P* < 0.05) with the increased SB supplementation. The activities of SOD, GSH-Px, CAT, and the concentration of MDA were determined at age of 14, 28, and 60 days. The former two measurements significantly decreased (*P* < 0.05), while the latter two measurements significantly increased (*P* < 0.05) over the course of the experiment (data not shown). No treatment × day interaction was observed (*P* > 0.05).

The supplementation with differing doses of SB in the present study did not influence (*P* > 0.05) the serum concentrations of IgA, IgG, or IgM in the calves during the whole experimental period, and no effects of day or treatment × day interaction was observed (Table 5).

## Discussion

### Sodium butyrate enhances feed utilization and average daily gain in calves before weaning

Dietary butyrate benefits young calves [17, 21, 29], but milk or milk replacer contains little butyrate. Indeed, the milk and MR used in the present study contained only 4.64 and 52.99 mg/L of butyrate, respectively. The amount of butyrate consumed from the milk and milk replacer by the calves in the present study was less than 0.7 g/d. Therefore, external butyrate needs to be supplemented to milk and milk replacer. Several studies reported that dietary supplementation with SB could enhance animal growth and stimulate the growth of duodenal mucosa in broiler chickens [30], stimulate the growth performance and feed intake in young pigs, especially before weaning [16], enhance the development of jejunal and ileal mucosa in formula-fed piglets [14], and improve the growth performance of young calves [12]. The positive effects of supplementation of liquid feeds with SB on the growth parameters observed in our study corroborate the previous studies and support the notion that butyrate supplementation is more effective when fed to dairy calves earlier rather than later [14, 16]. In newborn calves, solid feed intake largely depends on the development of the rumen, including the rumen tissue, rumen papillae, and the rumen microbiome [31, 32]. In the present study, we did not see any increase in feed intake attributable to SB supplementation. This is consistent with the reports by Hill et al. [15] and Vazquez-Mendoza et al. [33]. The ADG increased quadratically with the increasing SB dose, which concurs with the improved ADG previously observed in weaned calves supplemented with SB [21]. Although not reaching statistical significance, the SB supplementation also showed a linear trend in reducing the F:G ratio, and SB supplementation at 15 g/d decreased the F:G ratio by 8.3% throughout the feeding trial, and the F:G ratio were lower numerically in all the SB treatment groups than in the SB0 group. Such a magnitude is not appreciable, and future studies at the farm level can confirm the statistical and biological significance.

Several studies have reported different modes of action of SB supplementation in young animals. One study suggested that butyrate might enhance growth performance in young calves by improving feed digestibility [12], while another study proposed that in rats and pigs butyrate might enhance the absorption capacity of nutrients by increasing the depth of the crypts and the length of the small intestine villi, thus increasing the absorptive surface area [34]. In newborn calves, SB was shown to stimulate the development of the rumen [23] and small intestines [24] and enhance the maturation of the intestinal tract (including increased villus height and activities of digestive enzymes) [12]. The mode of action of SB supplementation may depend on the growth stage of calves. Future wholistic studies are needed to elucidate the underlying mechanisms by integrating transcriptomic and proteomic approaches coupled with morphological and histochemical methodologies to investigate the growth and development of the host, especially the digestive system, and meta-omic approaches to investigate the rumen microbiome.

### Sodium butyrate does not affect rumen fermentation in calves before weaning

Rumen fermentation starts at a very young age in calves, and VFAs can be found in their rumen from the second week of their lives [35]. In the rumen, butyrate confers multiple protective benefits, such as improving tight junctions, epithelial energy mobilization, and VFA absorption capacity [2]. Studies have shown that butyric acid could lower the rumen pH of calves [36], which can promote the GI colonization with beneficial bacteria [13]. However, our study showed that SB did not affect the rumen pH of the calves, probably because most of the SB bypassed the rumen together with the ingested liquid feed, and DMI (either total or starter) was not affected. Nevertheless, the rumen pH recorded in the present study should not have any detrimental effects on rumen development. The ruminal VFA concentrations, both total and individual, were not affected by the supplement with SB in this study. However, as demonstrated in other studies [23, 37, 38], SB supplementation via rumen cannulae might have enhanced the absorption of VFAs in the rumen due to the positive stimulation of the growth of rumen papillae [38]. Further research should investigate the mechanism by which SB enhances the production and absorption of VFAs. No effect of SB on the NH_3_-N concentration was observed in this study, which could reflect the balance of protein degradation and NH_3_-N uptake by rumen microbes for protein synthesis [39]. More research needs to be done to investigate the effect of SB supplemented in not only liquid feeds but also solid feeds on nitrogen utilization in calves.

### Sodium butyrate enhances the serum antioxidant capability in calves before weaning

Calving leads to oxidative stress, which can increase the formation of reactive oxygen species (ROS) and overwhelm the antioxidant systems of calves [40]. Reactive oxygen species, and reactive nitrogen species (RNS) to a lesser extent, can cause oxidative damages to tissues and overwhelm the body’s endogenous antioxidant capacity [41]. The antioxidative enzymes, such as SOD, GSH-Px, and CAT [42], are the essential components of oxidative stress defense systems in animals, including calves. In the present study, we evaluated how SB might affect the activities of those antioxidative enzymes and the concentration of MDA, a marker for oxidative stress, in the serum. The GSH-Px activity linearly increased with increasing SB levels, while the serum MDA concentration decreased linearly, indicating that the SB supplementation might have boosted the oxidative stress defense system while attenuating oxidative stress. A previous study showed that dietary SB increased the activity of SOD and decreased serum MDA concentration in chicken [43]. Using an IPEC-J2 cell model of piglets, Ma et al. [44] showed that SB could attenuate the oxidative stress in the intestinal mucosa and suggested that SB could improve the intestinal tight junction and decrease its permeability by improving antioxidant ability as one mechanism. Butyrate has also been shown to decrease the oxidative damages to human colorectal cells [45], reduce oxidative stress precipitated by colonic inflammation caused by cancer-induced destruction of the intestinal barrier [46], and alleviate oxidative stress induced by lipopolysaccharides in intestinal epithelial Caco-2 cells and colonic mucosa [47] and streptozotocin-diabetic rats [48]. The discrepancies between our study and the above studies with respect to the activity of SOD may be attributable to differences in the levels of SB and the species of animals used. Nevertheless, the increased GSH-Px activity and decreased MDA concentration among the calves supplemented with SB demonstrate the benefits of SB supplementation to help the calves in coping with the oxidative stress from which they suffered in their young lives.

### Sodium butyrate does not affect serum concentrations of IgA, IgG, or IgM in calves before weaning

As three important antibodies of the immune function of animals including calves, IgA, IgG, and IgM can protect animals and humans against a variety of pathogens and viruses, activate the complement system, regulate the antibody-dependent cell-mediated cytotoxicity, and improve animal’s immunity [49]. Butyrate has been found to have a profound impact on the immune system in humans and rodents [50]. Supplementation with SB also increased the number of IgA^+^ cells, which later increased the production of secretory IgA in the jejunum of piglets [51] and increased serum IgG concentrations in pigs [52]. In the present study, supplementation with SB in liquid feeds did not affect the serum concentrations of any of the three antibodies in the calves, which was in general agreement with the report that supplementation with SB in acidified milk did not affect the serum immunoglobulin concentration in calves [53]. The discrepancies between our study and the studies on other animal species might be attributable to differences in SB supplementation levels, methods of SB supplementation, and the animal species used. Further research is warranted to further investigate if butyrate modulates immune system development and function in calves using other immunological analyses than just analysis for the three Ig.

## Conclusions

Under the conditions of this study, SB supplementation in liquid feeds (milk and/or milk replacer) improved growth performance, feed efficiency, and antioxidant ability in pre-weaned dairy calves. We recommended 45 g/d as the optimal level of SB supplementation mixed into liquid feeds to improve the growth and antioxidant function of dairy calves before weaning. Farm-level studies involving large numbers of calves are needed to evaluate if SB can improve the growth and development of the rumen and intestine and animal health. Mechanistic studies using physiological, immunological, transcriptomic, and proteomic methodologies and technologies are also needed to elucidate how butyrate enhances growth and antioxidant function in calves before weaning.

## Data Availability

Not applicable.

## Abbreviations

GI: Gastrointestinal
SB: Sodium butyrate
SCFA: Short-chain fatty acid
DM: Dry matter
ADG: Average daily gain
BW: Body weight
MR: Milk replacer
DMI: Dry matter intake
F:G: Feed-to-gain
NH_3_-N: Ammonia nitrogen
VFAs: Volatile fatty acids
GSH-Px: Glutathione peroxidase
SOD: Superoxide dismutase
MDA: Maleic dialdehyde
IgA: Immunoglobulin A
IgG: Immunoglobulin G
IgM: Immunoglobulin M
